# An update on preclinical models of hereditary haemorrhagic telangiectasia: Insights into disease mechanisms

**DOI:** 10.3389/fmed.2022.973964

**Published:** 2022-09-29

**Authors:** Helen M. Arthur, Beth L. Roman

**Affiliations:** ^1^Biosciences Institute, Centre for Life, University of Newcastle, Newcastle, United Kingdom; ^2^Department of Human Genetics, School of Public Health, University of Pittsburgh, Pittsburgh, PA, United States; ^3^Heart, Lung, Blood and Vascular Medicine Institute, University of Pittsburgh, Pittsburgh, PA, United States

**Keywords:** HHT, arteriovenous malformation (AVM), endoglin (CD105), activin receptor-like kinase 1 (ACVRL1), BMP-SMAD signalling pathway, cell polarity and migration

## Abstract

Endoglin (ENG) is expressed on the surface of endothelial cells (ECs) where it efficiently binds circulating BMP9 and BMP10 ligands to initiate activin A receptor like type 1 (ALK1) protein signalling to protect the vascular architecture. Patients heterozygous for *ENG* or *ALK1* mutations develop the vascular disorder known as hereditary haemorrhagic telangiectasia (HHT). Many patients with this disorder suffer from anaemia, and are also at increased risk of stroke and high output heart failure. Recent work using animal models of HHT has revealed new insights into cellular and molecular mechanisms causing this disease. Loss of the *ENG* (HHT1) or *ALK1* (HHT2) gene in ECs leads to aberrant arteriovenous connections or malformations (AVMs) in developing blood vessels. Similar phenotypes develop following combined EC specific loss of SMAD1 and 5, or EC loss of SMAD4. Taken together these data point to the essential role of the BMP9/10-ENG-ALK1-SMAD1/5-SMAD4 pathway in protecting the vasculature from AVMs. Altered directional migration of ECs in response to shear stress and increased EC proliferation are now recognised as critical factors driving AVM formation. Disruption of the ENG/ALK1 signalling pathway also affects EC responses to vascular endothelial growth factor (VEGF) and crosstalk between ECs and vascular smooth muscle cells. It is striking that the vascular lesions in HHT are both localised and tissue specific. Increasing evidence points to the importance of a second genetic hit to generate biallelic mutations, and the sporadic nature of such somatic mutations would explain the localised formation of vascular lesions. In addition, different pro-angiogenic drivers of AVM formation are likely to be at play during the patient’s life course. For example, inflammation is a key driver of vessel remodelling in postnatal life, and may turn out to be an important driver of HHT disease. The current wealth of preclinical models of HHT has led to increased understanding of AVM development and revealed new therapeutic approaches to treat AVMs, and form the topic of this review.

## Introduction

Hereditary haemorrhagic telangiectasia (HHT) is an inherited vascular disease characterised by multiple localised abnormal connections between an artery and a vein. These vascular lesions place HHT patients with an increased risk of anaemia, stroke, abscess, and high-output heart failure. The majority (>85%) of HHT patients are heterozygous for loss of function (LOF) mutations in the endoglin (*ENG*, HHT1) or activin A receptor like type 1 (*ALK1*, HHT2) genes (1, 2), whilst a minority (∼5%) carry mutations in the *SMAD4* gene and show a combined juvenile polyposis and HHT phenotype (3). A very rare group of patients with an HHT-like phenotype map to mutations in *BMP9* (also known as *GDF2*) (4, 5). The generation of animal models with mutation in these (and related) genes has revealed significant insights into disease mechanisms and pathology that have also opened up major opportunities for drug screening to treat HHT. This review will discuss recent advances in this field, focussing on new insights into the molecular mechanisms that underpin localised aberrant blood vessel remodelling in HHT.

## Endoglin promotes BMP9/10 signalling *via* ALK1 in endothelial cells

**FIGURE 1:**
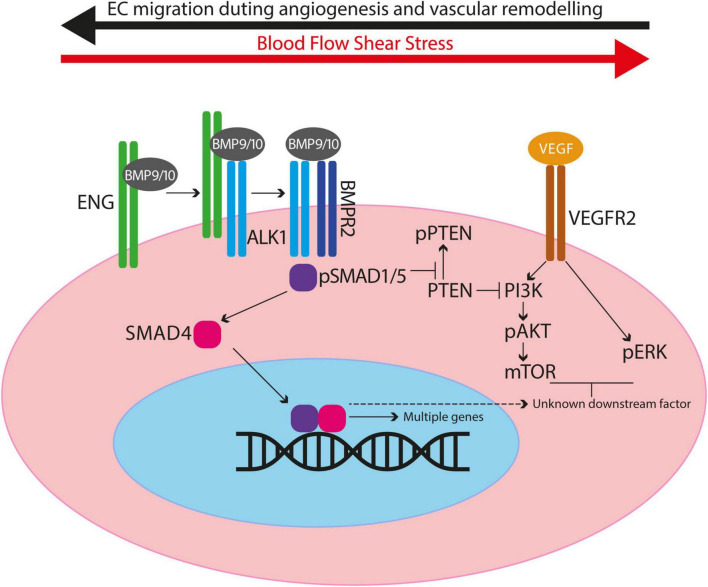
Diagram to illustrate crosstalk between BMP9/10 signalling and VEGF signalling in endothelial cells. ENG can act as a reservoir of BMP9/10 ligand on endothelial cells and blood flow enhances ALK1/ENG interaction. ENG is displaced when the type II receptor (e.g., BMPR2) joins the protein complex. Type II receptor phosphorylates ALK1 thereby activating its kinase activity to phosphorylate SMAD1/5. Phospho-SMAD1/5 (pSMAD1/5) interacts with SMAD4 to migrate to the nucleus and act as transcription factors to regulate expression of multiple genes. BMP9/10 signalling prevents inactivation of PTEN, consequently inhibiting PI3K activity and reducing downstream AKT activation. Thus in the absence of BMP9/10 signalling there is increased PI3K activity leading to increased EC proliferation and survival. Also, there is evidence for BMP9/10 signalling events that inhibit VEGF signalling outcomes after a minimum of 2 h, consistent with downstream gene expression (see main text).

Endoglin is a type III co-receptor for the ALK1 serine/threonine kinase signalling receptor in vascular endothelial cells (ECs), and the signalling pathway is summarised in Figure 1. Circulating ligands bone morphogenetic protein (BMP) BMP9 and BMP10 bind to a reservoir of transmembrane ENG, which is present as a disulphide-linked dimer on the surface of ECs (6). Ligand is then transferred to ALK1 to form a transient BMP9/10-ENG-ALK1 protein complex, and ENG is released to enable a type II BMP receptor (BMPR2, ACTRIIA/B) to join, forming the BMP9/10-ALK1-BMPR2 signalling complex (6, 7). The type II receptor phosphorylates ALK1, which in turn phosphorylates SMAD1/5 transcription factors that can then bind to SMAD4 and move to the nucleus to regulate gene expression. In this way, transmembrane ENG acts as an accessory protein that enhances EC transcriptional responses to circulating BMP9/10 ligands. The extracellular domain of ENG protein can also be released from the EC surface *via* a protease mediated mechanism (8), an event that may reflect endothelial dysfunction. This soluble form of ENG (sENG) can bind to ligand and, rather than behaving as a ligand trap, the BMP9-sENG complex can promote BMP9 signalling by interacting with endothelial transmembrane ENG (6). High levels of circulating sENG in human pregnancy are associated with pre-eclampsia (9) and transgenic mice expressing high levels of circulating sENG develop increased arterial blood pressure (10). These findings suggest a role for sENG in blood pressure regulation, although the molecular mechanisms are not understood.

The preponderance of evidence demonstrates that ENG promotes but is not absolutely essential for ligand-mediated ALK1 signalling (11–14), but the underlying mechanism is not fully understood. BMP9 and BMP10 growth factor dimers (GFDs) bind to the ectodomains of both ENG and ALK1 with picomolar to low-nanomolar affinity (15–18), so it is unlikely that ENG simply functions as a high-affinity binder. There are a number of possible ways in which ENG may enhance ALK1 signalling in response to BMP9/10 ligands. The long ectodomain of ENG may (i) be important in reaching into the blood vessel lumen to capture circulating ligand and/or (ii) provide a transient store of ligand on the EC surface (6) and/or (iii) enhance ligand affinity due to increased avidity of its dimeric form (19). ENG may also more efficiently displace (non-covalently bound) prodomains from GFD than ALK1 protein (6, 20) to enhance signalling through ALK1. Interestingly, a full-length, uncleaved and therefore latent form of BMP10 is detected in blood (21) and it is also possible that ENG somehow plays a facilitating role in its activation.

The role of the intracellular C-terminal domain of ENG in ALK1 signalling in ECs is less well understood. This domain has been shown to interact with zyxin and zyxin-related protein 1 (ZRP1) to favour assembly of actin stress fibres and inhibit EC migration (22, 23), but whether it is required for ALK1 signalling is unclear.

## Vascular malformations in hereditary haemorrhagic telangiectasia

**FIGURE 2:**
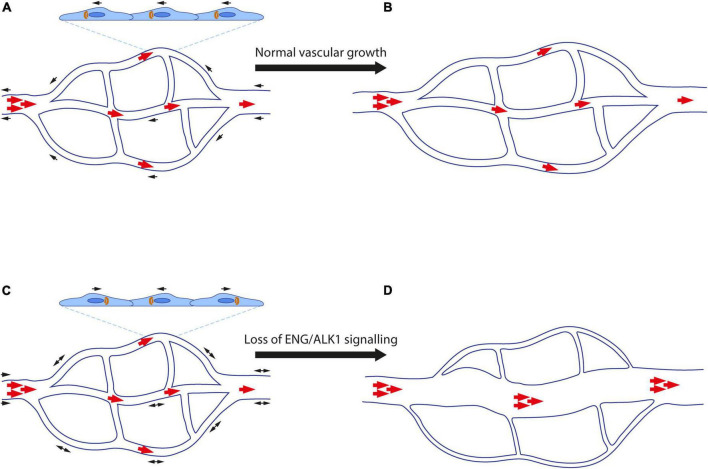
Abnormal endothelial cell migration contributes to formation of arteriovenous malformations. **(A)** Endothelial cells (ECs) polarise in response to blood flow (red arrows) and migrate (black arrows) against the direction of flow. Golgi bodies (orange) are aligned with respect to the nucleus (dark blue) in the direction of EC migration. **(B)** ECs proliferate and elongate in line with blood flow as the vasculature remodels and matures. High blood pressure is retained in the artery, whilst the vascular architecture enables lower blood pressure to reach capillaries and veins. **(C)** Loss of ENG/ALK1 signalling leads to loss of EC polarisation, reduced directional cell migration. Stalled ECs continue to proliferate resulting in an AVM. **(D)** Blood flow favours the major channel created by the AVM, such that blood reaches the veins at abnormally high pressure, whilst the remaining capillary plexus atrophies in the absence of flow.

ENG/ALK1 signalling is essential for maintaining normal vascular architecture during periods of vessel growth and remodelling. As a result, loss of endothelial ENG and ALK1 signalling results in arteriovenous malformations (AVMs), which are abnormal direct connections between an artery and a vein (Figure 2).

Where present, AVMs allow arterial blood to bypass the normal capillary bed and reach the vein at abnormally high pressure. Small superficial AVMs in the skin, mouth, nose and gastrointestinal tract are known as telangiectases, and those found in the mucosal tissues are particularly prone to rupture and bleeding, with severe cases leading to anaemia. Large AVMs in the lung lead to cyanosis as blood can bypass the oxygenating alveolar microvasculature, and even small AVMs increase risk of stroke and brain abscess due to reduced filtering of micro-thrombi and bacteria by pulmonary capillaries. In addition, cerebral AVMs carry an intrinsic risk of haemorrhagic stroke, whilst multiple vascular malformations in the liver can increase venous return to the heart that may result in high output heart failure. Thus, HHT patients can suffer multiple pathologies, depending on the frequency and location of “micro” and “macro” AVMs.

Given that ENG is an accessory receptor that facilitates but is not essential for ALK1 signalling, one might expect almost complete phenotypic overlap between HHT1 and HHT2 (perhaps with ALK1/HHT2 patients showing more severe disease). However, this is not the case and there are clear genotype/phenotype correlations in HHT that are not understood. For example, ENG/HHT1 patients are more likely to have lung AVMs, brain AVMs and earlier onset nosebleeds compared to ALK1/HHT2 patients. By contrast, ALK1/HHT2 patients are more likely to have liver AVMs and consequent high output heart failure (24). In this latter context, high local BMP9 concentrations (BMP9 is made in the liver) could make hepatic ECs less dependent on ENG and more dependent on ALK1 activity.

It is possible that ENG and ALK1 interact with different partner proteins in different vascular beds to elicit differential downstream signalling. There is evidence from *in vitro* interaction assays that ENG and ALK1 each binds some unique proteins in addition to a common set of proteins (25). However, these findings clearly require confirmation in living tissues.

Thus, although it is generally recognised that AVMs result from deficient ENG/ALK1 signalling, better understanding of the underlying molecular mechanisms remains a goal of ongoing investigations.

## Mouse models of hereditary haemorrhagic telangiectasia

As previously reviewed (26, 27), complete loss of *Eng* or *Alk1* leads to embryonic lethality and the first mouse models of HHT1 and HHT2 were based on *Eng*^+/−^ and *Alk1*^+/−^ mice, corresponding to the patient genotype at these loci. However, taken overall, and compared with the disease observed in more recent models, reported features of HHT in these heterozygous preclinical models are less penetrant and less severe. More recent preclinical mouse models in which the Cre/Lox system is used to delete *Eng* or *Alk1* demonstrated that EC-specific loss of either gene robustly leads to AVMs during angiogenesis. Furthermore, a very comparable AVM phenotype appears in the developing neonatal retinal vasculature following (i) EC-specific loss of *Smad4* (28–30), (ii) EC-specific dual loss of *Smad1* and *Smad5* (31), or (iii) dual sequestration of BMP9 and BMP10 protein using a transmammary-delivered antibody approach (32). As the blood vessel pathology is so similar in the neonatal retina across all these genetic mouse models, these findings are consistent with a central role for ENG in the signalling pathway BMP9/10-ENG-ALK1-SMAD1/5-SMAD4 that functions in ECs to protect the developing vasculature against AVMs.

A similar endothelial-specific role for this pathway in preventing AVMs is supported by adult models of HHT (27). However, in adult life, where the majority of blood vessels are quiescent, AVMs do not appear to form without an angiogenic trigger. For example, loss of endothelial *Eng* in adult mice leads to peripheral AVMs in the vasculature supplying the non-capsulated cartilage of the pubic symphysis, which expresses high endogenous levels of VEGF (33). The AVMs result in increased venous return to the heart, cardiac enlargement, and high-output heart failure. Similarly, endothelial loss of *Alk1* or *Eng* in adult mice leads to skin AVMs only after a secondary insult such as dermal wounding (34). In this model, spectral visualisation of blood vessel remodelling in a window chamber system has revealed details of AVM initiation and progression. First, small AV shunts are observed, followed by enlargement of draining veins and feeding arteries (likely due to hemodynamic cues), and adjacent capillaries regress as the AV shunts mature (34, 35). In adult mice, brain AVMs develop with focal deletion of *Eng* or *Alk1*, but require exogenous VEGF stimulation (36, 37). Thus, ENG and ALK1 function in ECs (likely *via* the same BMP9/10-ENG-ALK1-SMAD1/5-SMAD4 pathway) to enable normal vascular remodelling of adult blood vessels in response to angiogenic stimuli.

## Cell autonomy of ENG/ALK1 signalling in arteriovenous malformation prevention

There is general consensus in the literature that AVM prevention requires ENG/ALK1 signalling in the vascular endothelium: in mice, both genes are predominantly expressed in ECs, and pan-EC deletion generally phenocopies global deletion, as discussed above. Although a functional requirement for this pathway in vascular support cells has been reported (38, 39), the *Tagln* (also known as *SM22*) Cre driver used in these experiments deletes in both vascular smooth muscle cells (VSMCs) and select ECs (40), so does not rule out an endothelial requirement for ENG/ALK1 signalling. Furthermore, *Alk1* and/or *Eng* deletion *via* glial- and pericyte-specific *NG2*-driven Cre or SMC-specific *Myh11*-driven Cre failed to generate AVMs (34, 36). *Eng* requirements have also been examined in macrophages (using *LysM*-Cre) revealing no role in AVM prevention, but instead a role in monocyte-macrophage differentiation and immune response to infection (36, 41).

A number of studies suggest that “non-resident” ECs carrying mutations in *Eng* or *Alk1* can contribute to vascular lesions in HHT. Transplantation of bone marrow cells from *Eng*^+/−^ mice into wild type mice led to capillary dysplasia in VEGF-stimulated brain regions (42). However, the identity of the malformation-promoting cells in this experiment was unclear. More recently, using a more sophisticated experimental design, fluorescently labelled *Alk1*-null bone marrow cells transplanted to wild type mice contribute as ECs to the vasculature of VEGF-stimulated brain AVMs and intestinal AVMs (43). Important evidence for contribution of “non-resident” ECs to vascular lesions also comes from HHT patients following liver transplant from a donor of the opposite sex, and later recurrence of hepatic HHT disease (44). Specifically, in cases where female HHT patients have received a liver from a male non-HHT donor, female (XX) ECs can be observed within hepatic vascular lesions. The source of circulating EC progenitors is unclear and the process is slow, as disease recurrence happens only after a number of years. Additional studies are required to unequivocally identify the EC progenitor cells that contribute to these AVMs, and whether they originate from bone marrow or other tissue specific niches.

## Somatic mutations and arteriovenous malformation mosaicism in hereditary haemorrhagic telangiectasia

Hereditary haemorrhagic telangiectasia is an autosomal dominant, loss-of-function disease, but physiological vessel development appears to be largely normal and vascular lesions are limited to relatively small areas. This points to widespread haplo-sufficiency of the single functioning *ENG* or *ALK1* allele in HHT1 and HHT2 patients, respectively. Based on animal model data, only when the remaining functioning allele is lost in ECs (e.g., due to a second genetic hit) would wounding or some other angiogenic trigger be a likely risk factor for AVMs. Indeed, recent evidence supports the second somatic hit model in patients: biallelic second genetic hits have recently been confirmed in HHT1 and HHT2 patient dermal telangiectases (45). Although endothelial specificity of these mutations and the presence of second genetic hits in larger AVMs awaits further investigation, this initial finding in HHT patients has brought clinical and preclinical HHT studies into close alignment. Specifically, homozygous loss of endothelial *Alk1* or *Eng* is likely to be an essential prerequisite for AVM formation. Importantly in this regard, mosaic biallelic loss of either *Eng* or *Alk1* gene function is sufficient to cause retinal AVMs in mouse models of HHT (13, 37, 46). In mosaic *Alk1* mutants, shunts reportedly consist only of mutant cells (13), whereas in mosaic *Eng* mutants, shunts contain both mutant and wild type cells (46). Regardless of this apparent difference, these findings in HHT patients and mouse models suggest that mosaic loss-of-function mutations in HHT genes may govern lesion location.

## ENG/ALK1 signalling as a mediator of endothelial cell shear stress response

While it is clear that loss of ENG/ALK1 signalling in ECs leads to AVMs, how this pathway governs EC behaviour is still being investigated and increasing evidence points to a key role in the shear stress response. It is well-known that ECs transduce hemodynamic force into biochemical signals that govern EC behaviour and are critical for vascular remodelling. For example, laminar shear stress promotes EC elongation along the flow axis, dampens EC proliferation, and is required for EC migration against flow (47–50). Experiments addressing each of these aspects in the context of disrupted ENG/ALK1 signalling are discussed below.

### ENG/ALK1 signalling controls flow-migration coupling

Zebrafish *alk1* mutant embryos develop cranial AVMs at a predictable time (36–48 h post-fertilisation), and location (beneath the brain) (51). As such, these externally fertilised, optically clear embryos, in which vascular development can be evaluated in real time by imaging EC-specific fluorescent transgene expression, are ideal for assessing EC behaviours that lead to AVMs. Time-lapse confocal imaging of wild type embryos revealed that arterial ECs lining patent cranial vessels close to the heart migrate against the direction of blood flow in an *alk1*-dependent manner. In *alk1* mutants, decreased EC migration against blood flow and increased EC migration in the direction of blood flow result in distal enlargement of these arteries (52). Furthermore, aberrant EC migratory behaviour in the absence of blood flow resembles that seen in *alk1* mutants, suggesting that Alk1 signalling is important in transducing a blood flow-based signal that directs EC migration (52). However, this migratory defect alone does not directly cause AVMs. Instead, AVMs develop downstream of these enlarged vessels and represent arteriovenous connections that are pruned in wild type embryos (53) but retained in *alk1* mutants (54). Importantly, AVMs in *alk1* mutants form only in the presence of blood flow and often connect arterial and venous vessels that do not express *alk1*. Together, these data suggest that AVMs represent a maladaptive response to altered hemodynamic force due to enlargement of upstream *alk1*-dependent arteries (54).

In concordance with the zebrafish *alk1* model, impaired “flow-migration coupling” was subsequently observed in mouse retinas where ECs are null for *Alk1* or *Eng* (46, 55). During development of the mouse neonatal retina, the vasculature forms as venous ECs migrate against the direction of blood flow from growing veins to contribute to the growth of newly forming tip cells, capillaries, and arterial vessels (56, 57). Recent work using genetic mouse models shows that *Eng* and *Alk1* are important for this process, and loss of *Eng* or *Alk1* from ECs leads to reduced EC migration against the direction of blood flow (46, 55). This defect results in an accumulation of “stalled” ECs within interconnecting capillaries, leading to their enlargement to form abnormal arteriovenous connections, resembling AVMs. These AVMs are maintained by blood flow and can further enlarge, simultaneously stealing blood from adjacent capillaries that then regress.

A defect in venous to arterial EC migration is consistent with the finding that *Alk1* depletion from arterial ECs does not result in AVM formation in the mouse neonatal retina, whereas loss of either *Eng* or *Alk1* from venous and capillary ECs is sufficient to drive AVM pathology (55, 58). However, for several reasons, it seems premature to conclude that Alk1 signalling is not required in arterial ECs to protect against AVM formation. First, outside of the mouse retina, *Alk1* predominates in arterial over venous ECs in mice and in zebrafish (51, 59). Second, within the retina, venous-derived ECs actually trans-fate to arterial ECs (56, 57). Third, under the experimental conditions used to determine that *Alk1* is not required in arterial ECs (55), the *BMX-Cre* transgene deleted *Alk1* in retina only in proximal arterial ECs, and activity outside the retina was not assessed. Assessment of EC subtype requirements for *Alk1* and *Eng* outside of the neonatal mouse retina is required to further clarify this issue.

### ENG/ALK1 signalling controls endothelial cell planar polarity in response to blood flow and shear stress

An important factor in the directional migration of ECs is their alignment with blood flow and their planar cell polarity: generally, the EC Golgi localises upstream of the nucleus, suggesting that ECs are poised to migrate against the direction of blood flow, with a higher degree of polarisation in arterial versus venous ECs (60–62). As first shown in developing hindbrains of mouse embryos with ECs deficient in *Smad1* and *Smad5* (63), and subsequently in coronary arteries deficient in *Smad4* (64) and neonatal retinas with ECs deficient in *Eng* or *Alk1* (46, 55, 63), ECs without the ability to respond to BMP signalling lose planar cell polarity. Furthermore, aortic ECs enlarge and fail to elongate along the blood flow axis in zebrafish *eng* mutant embryos; although Golgi-nuclear polarisation and EC migration were not addressed in this work, the defect in EC size and directional elongation suggests a similar loss of EC polarity (65). In addition, mechanosensitive SMAD1/5 signalling downregulates expression of the gap junction protein connexin37, which is required for directional cell migration against flow (66). Taken together, these findings support the notion that EC polarity is established in response to mechanotransduction signals from shear stress informing the cell of the directionality of blood flow. Thus, loss of EC polarisation in the absence of ENG-ALK1-SMAD1/5-SMAD4 signalling may underlie the loss of directionality of EC migration in response to blood flow, which in turn leads to AVMs (Figure 2). However, simultaneous analysis of planar polarisation and EC migration in real time in HHT models is required to firmly test this hypothesis.

### ENG/ALK1 signalling may dampen endothelial cell proliferation in response to flow

In neonatal mice, retinal ECs in the absence of *Eng*, *Alk1*, or *Smad4* show increased cell proliferation within AVMs (29, 30, 46, 67, 68). In the *Smad4* model, EC proliferation is increased in all vessel types regardless of proximity to an AVM, and clonal expansion of mutant *Alk1* ECs contributes directly to AVM enlargement (43). As shear stress promotes EC quiescence, increased cell proliferation may result directly from lack of a functional BMP9/10-ENG-ALK1-SMAD1/5-SMAD4 signalling pathway (13). Consistent with this hypothesis, circulating BMP9 and BMP10 promote vascular quiescence (69, 70) and there is growing evidence in ECs for altered cell autonomous VEGF signalling responses in the absence of ENG/ALK1 signalling (see below). However, enhanced EC proliferation may also be a secondary effect of AVMs where there is local bypass/loss of capillaries essential for tissue oxygenation. The resultant local regions of hypoxia would lead to release of VEGF, which also drives proliferation of neighbouring ECs leading to increased vascular sprouting (46).

### Shear stress potentiates ALK1 signalling

There is considerable evidence that ENG/ALK1 signalling is important in transducing hemodynamic cues in ECs. In zebrafish embryos, the initiation and persistence of *alk1* expression requires blood flow (54), and in mice, *Alk1* expression is increased in vessel segments experiencing higher flow (58, 59). Additionally, shear stress potentiates BMP9-mediated ALK1 signalling by enhancing the physical interaction between ALK1 and ENG proteins, thereby lowering the EC_50_ of BMP9 by approximately 17-fold (13). As sphingosine-1-phosphate (S1P)/S1PR1 signalling enhances *ENG* cell surface expression and BMP9-mediated ALK1 signalling (71, 72), it is interesting to speculate that shear stress-mediated activation of S1P/S1PR may be a key player in shear stress-induced enhancement of ALK1 signalling. Furthermore, although ALK1 does not seem to interact with the classical shear stress-responsive KLF2 pathway (13, 54), it does transduce flow signals to regulate expression of some flow-responsive genes, including *cxcr4a* and *edn1* (54).

## Role of vascular mural cells in hereditary haemorrhagic telangiectasia-associated arteriovenous malformations

As reviewed above, a series of genetic studies suggests that any ENG or ALK1 protein that may be present in VSMCs or pericytes has no obvious role in protecting against AVM formation (27). However, cross talk between ECs and VSMCs and pericytes is clearly disrupted when ENG or ALK1 is missing from ECs. Pericyte coverage of murine retinal vessels is reduced in the absence of endothelial *Alk1*, and a similar deficiency in mural cell coverage occurs with focal *Alk1* depletion in the mouse brain (68, 73). Complete or partial loss of *Eng* in mice leads to reduced VSMC coverage and destabilised vessels (74–77). Furthermore, ENG has been shown to interact with β1 integrin to regulate adhesion of mural cells to the endothelium (78). However, it seems unlikely that lack of mural cell coverage is a primary cause of AVMs, as AVMs in *alk1* mutant zebrafish develop prior to the onset of mural cell recruitment in wild type animals (52, 54, 79). Nevertheless, the flow-dependent recruitment of pericytes to cultured ECs is clearly dependent on *ALK1* and *ENG* (13), and shear stress is important for maintaining VSMC coverage of small blood vessels (80). Accordingly, impaired shear stress mechanotransduction may underpin the failure in EC-VSMC crosstalk required to maintain vascular stability and lead to vessel fragility in HHT. This interpretation is in line with histological analysis of sporadic cerebral AVMs, in which reduced pericyte coverage is associated with AVM instability and vascular fragility (81). Normally, ECs and VSMCs have an intimate connection involving gap junctions, and paracrine TGFβ signalling from EC to adjacent mural cells promotes activation of the intramural ALK5 pathway to promote smooth muscle differentiation and maturation which results in increased vessel stability (82). However, loss of ENG leads to reduced TGFβ1 bioavailability (74, 75). Taken together, these findings led to a better understanding of how thalidomide can protect against bleeding in HHT, due to its ability to increase platelet-derived growth factor (PDGF) signalling between ECs and VSMCs and thereby compensate for lost TGFβ1 to increase vessel stability (76, 83). Current clinical research efforts focus on less neurotoxic versions of thalidomide to reduce epistaxis and GI bleeding in HHT.

## Redundancy and compensation within the BMP9/10-ENG-ALK1-SMAD1/5-SMAD4 pathway

There are important differences in the roles of growth factors BMP9 and BMP10 in development and disease. For example, BMP10, but not BMP9, is essential for cardiac development (84), whereas BMP9, but not BMP10, dampens tumour vessel growth (85). However, with respect to vascular development and AVM prevention, these proteins were initially thought to be genetically redundant ALK1 ligands. For example, BMP9 and BMP10 activate the same cohort of genes when applied to human pulmonary arterial ECs (20, 86), and several studies have reported that loss of both proteins by genetic means and/or blocking antibodies is required to generate vascular remodelling defects and AVMs in mouse models (32, 86–88). However, it should be noted that although postnatal retinal AVMs develop following combined treatment with BMP9 and BMP10 blocking antibodies, the effect of each antibody alone has not been reported. Moreover, although no phenotype was observed in *Bmp10* conditional knockouts (deleted at 2 months and assayed at 5 months), deletion was incomplete, with significant residual expression in liver and heart and detectable protein in plasma (88).

The situation appears clearer in zebrafish, where *bmp10* has emerged as the only necessary Alk1 ligand for AVM prevention. Zebrafish *bmp9* mutants develop no overt blood vessel defects or AVMs (similar to murine *Bmp9* mutants), whereas zebrafish embryos carrying homozygous null mutations in duplicate *BMP10* paralogs, *bmp10* and *bmp10-like*, develop embryonic lethal cranial AVMs identical to those documented in *alk1* mutants (89, 90). The presence of two *BMP10* paralogs in zebrafish allowed for later assessment of juvenile and postnatal phenotypes; surprisingly, whereas *bmp10-like* mutants develop no phenotype, *bmp10* mutants display vascular malformation in the skin and liver and high-output heart failure, with variable age of onset, penetrance, and expressivity (90), reminiscent of HHT. This phenotype is also similar to that seen in adult *eng* mutant zebrafish (65, 91), consistent with a critical role for the BMP10-ENG-ALK1 pathway in protecting against vascular malformations. Notably, the tissue/cellular source of Bmp10 appears to be important. Zebrafish *bmp10-like* is expressed in cardiomyocytes whilst *bmp10* is produced in endocardium and liver (89, 90). As *bmp10-like* mutants have no phenotype, whereas *bmp10* mutants do show AVMs, this points to Bmp10 from endocardium and/or liver as the key ligand in AVM prevention. This hypothesis is supported by evidence from mouse models: murine BMP10 is expressed strongly in the heart, but also in the liver (92), and myocardial-specific deletion of murine *Bmp10* does not generate AVMs (39). Thus, liver-derived BMP10 may be the critical circulating ligand in protecting against AVMs in HHT.

While additional studies are required to sort out the overlapping and unique requirements for BMP9 and BMP10, redundancy among receptor-specific SMADs is somewhat clearer. As mentioned above, endothelial loss of both *Smad1* and *Smad5* genes are required before mouse neonatal retinal AVMs form (31), suggesting redundancy of function between these two SMAD proteins in ECs. ALK1 might also phosphorylate SMAD9 (formerly known as SMAD8) and mutations in *Smad9* are associated with defective pulmonary vascular remodelling in mice (93) and are a rare cause of pulmonary arterial hypertension in humans (94). However, *Smad9* cannot compensate for loss of *Smad1* and *Smad5* with respect to AVM development in mice, and there is no documented association of *SMAD9* with HHT.

Loss of endothelial *Alk1* leads to reduced endothelial ENG protein expression (68), so it is of interest to know whether the *Alk1* mutant vascular phenotype results from loss of ALK1, ENG or both proteins. As expected, over-expression of human ENG (hENG-OE) can rescue the retinal AVM phenotype in *Eng*-iKOe mutants, consistent with the recessive nature of this disease (46). However, hENG-OE cannot rescue the vascular defects (wound-induced dermal AVMs and neonatal retinal AVMs) that occur in the absence of endothelial ALK1 protein in *Alk1*-iKOe mouse mutants (14). By contrast, *ALK1-OE* can rescue *Eng*-iKOe vascular defects (14). This finding is consistent with the role of ENG protein as an accessory receptor required to assist BMP9/10 ligand access to ALK1 *in vivo*, a role that appears to become redundant when excess ALK1 protein is present.

## Loss of ENG/ALK1 leads to altered vascular endothelial growth factor signalling responses in endothelial cells

As AVMs in HHT require the combination of genetic deficiency in ENG/ALK1 signalling with an angiogenic trigger, there has been considerable interest in the role of VEGF, the “master” regulator of angiogenesis. There are a number of ways in which cross talk between BMP9/10-ENG/ALK1 signalling and VEGF signalling regulates EC responses (95; Figure 1). BMP9/10 signalling prevents inactivation of PTEN, an inhibitor of the PI3K/AKT pathway. Thus, reduced BMP9/10 signalling leads to PI3K/AKT hyperactivity and disruption of normal EC responses. Consistent with this, ECs with loss of *Eng* or *Alk1* show increased activation of the PI3K/AKT pathway in response to VEGF stimulation compared with control ECs (46, 87, 96). Moreover, biopsies of cutaneous telangiectases from patients with HHT2 show increased PI3K activation (96, 97). Furthermore, there is evidence that BMP9/10 signalling inhibits VEGF signalling through downstream mediator(s) that remain to be identified (96). In consequence, loss of BMP9/10 signalling leads to an untempered EC response to VEGF such as increased cell proliferation that would contribute to AVM formation. Targeting VEGF signalling or key players in VEGF downstream signalling pathways (pERK, PI3K/mTOR) can protect against AVM development and progression in (i) *eng* null zebrafish embryos (91), (ii) developing retinal vasculatures of *Eng-iKOe, Alk1-iKOe*, and BMP9/10 depleted neonatal mice (46, 87, 98), and (iii) in wounded skin of *Alk1* deficient adult mice (99). Furthermore, combined low dose targeting of pERK and mTOR in *eng* mutant zebrafish protects against development of the HHT phenotype, suggesting synergy between these two pathways (91). However, a major limitation of targeting VEGF signalling during developmental angiogenesis in these neonatal mouse and zebrafish embryo HHT models is that blocking VEGF signalling inhibits progression of angiogenesis itself, making outcome analyses challenging to dissect. Therefore, the outcomes are clearer in adult models of HHT. Here, anti-VEGFR2 therapy reduces AVMs in *Eng-iKOe* mice (33) and anti-VEGF protects against wound induced AVMs in the global *Alk1-KO* mouse (99). The importance of VEGF in AVM progression is also consistent with the finding that *Alk1*^+/–^ mice show reduced release of soluble VEGFR1 (sVEGFR1) and circulating levels of sVEGFR1 are also reduced in HHT2 patients (100). As circulating sVEGFR1 represents a molecular sink for VEGF, reduced levels are expected to increase availability of VEGF to drive vascular malformations.

A recent study revealed increased VEGFR2-integrin interaction in *ALK1*-deficient ECS that leads to enhanced downstream YAP/TAZ nuclear translocation (55). On the one hand this work highlights the complexity of VEGFR2 downstream signalling changes in the absence of ALK1, whilst also opening up the possibility that integrin and/or YAP/TAZ signalling blockers may represent new potential targets to prevent vascular malformations in HHT.

Importantly, the anti-VEGF inhibitor bevacizumab reduces the risk of cardiac disease in HHT patients with liver vascular malformations and reduces nosebleeds from nasal telangiectases (101, 102), making VEGF one of the most significant drug targets for patient therapy, despite the risks of significant side effects.

## Inflammation and hereditary haemorrhagic telangiectasia

Early electron microscopy assessment of dermal telangiectases in HHT reported a notable leukocyte presence, primarily lymphocytes, pointing to an inflammatory component of these lesions (103). However, this observation does not inform whether lymphocytes have a causal vascular injury effect, or whether they represent a later adaptive immune response to a pre-existing vascular lesion. Nevertheless, inflammation has long been suspected as a trigger driving AVM formation in HHT (104). An interesting inflammatory model of HHT is *Alk1*^+/−^ mice in which the trachea are infected with bacteria to produce airway inflammation resulting in the formation of telangiectases (100). Telangiectases are reduced following treatment with VEGFR2 antibodies, in line with the requirement for an angiogenic trigger for their development.

Endothelial Eng expression is induced during inflammation and wound healing (105) and plays an important integrin-mediated role in leukocyte adhesion and extravasation (106, 107). Thus, reduced ENG protein in HHT1 may delay leukocyte extravasation and impair tissue repair. It is also possible that ENG plays a cell autonomous role within key inflammatory cells in inflammation and HHT disease. ENG is expressed on activated monocytes, raising the possibility that reduced ENG levels in HHT1 patients affects the native immune response. Mouse brain AVMs following deletion of *Eng* globally or *Alk1* focally show an increased monocyte infiltrate (108). Reduced ENG protein expression on monocytes from HHT1 and HHT2 patients lowers their capacity for recruitment to an SDF1 stimulus, which is released at the site of tissue injury (109–111). Thus HHT patients might be expected to have an impaired or delayed immune response to infection or tissue injury. In support of this notion, mice lacking ENG in macrophages show a compromised immune response to opportunistic infections (41), and *Eng*^+/–^ mice exhibit impaired recovery from inflammatory colitis (112) as well as impaired cardiac repair following myocardial infarction (110). A potential further link to inflammation is evidence that polymorphisms in the gene encoding TNFα converting enzyme (*ADAM17*) are associated with pulmonary AVM incidence in HHT1 patients (113). Inflammation also leads to loss of the endothelial glycocalyx, and inflammatory cytokines such TNFα trigger events leading to cleavage of the extracellular domain of endoglin (114). As baseline levels of ENG protein are 50% of normal in HHT1, then cleavage of the extracellular domain of ENG during inflammation may cause this to transiently drop below the levels required to maintain the normal vascular architecture (67). Despite these interesting data, the role of inflammation in HHT remains an understudied field of research.

## Discussion

**FIGURE 3:**
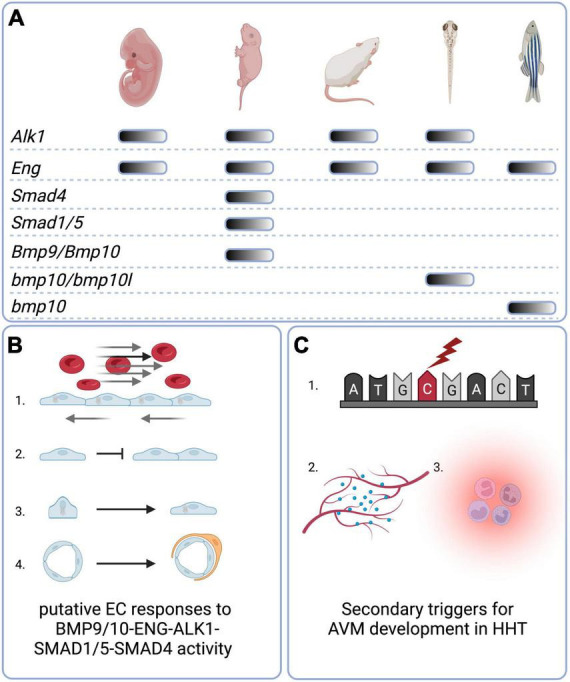
Graphical summary. **(A)** Animal models of HHT-like vascular lesions. Mouse genetic models include global embryonic models as well conditional embryonic, neonatal, and adult models. For BMP9 and BMP10, a neonatal “immunoblocked” model is also available. Zebrafish genetic models are global nulls that generate embryonic and/or adult phenotypes. **(B)** Putative direct or indirect EC responses to BMP9/10-ENG-ALK1-SMAD 1/5-SMAD4 activity include (1) Golgi-nuclear polarisation and migration against the direction of blood flow; (2) inhibition of proliferation; (3) elongation and decrease in overall cell size; (4) mural cell recruitment and adhesion. **(C)** Secondary triggers for AVM development in HHT include (1) somatic mutations in the wild type copy of the germline-mutated gene; (2) angiogenic triggers such as VEGF; (3) inflammation. Created with Biorender.com.

Recent findings in animal models of HHT have significantly contributed to our understanding of the pathobiology of HHT disease in several ways (as summarised in Figure 3). First, these studies reveal the importance of all members of the canonical signalling pathway BMP9/10-ENG/ALK1-SMAD1/5-SMAD4 in prevention of AVMs. The lack of reported HHT patients carrying mutations in all of these genes is likely due to the fundamental importance of these genes in embryonic development. On the other hand, polymorphic variants in genes affecting the BMP9/10 pathway or the VEGF pathway may be important modifiers of the HHT1/2 clinical phenotypes.

Second, in terms of key molecular mechanisms in AVM formation, recent studies highlight the importance of endothelial ENG/ALK1 signalling in EC mechanotransduction in response to blood flow as a regulator of directional EC migration. Furthermore, even mosaic genetic defects in the ENG/ALK1 signalling pathway of ECs are sufficient to result in AVMs, suggesting that a subset of misdirected and/or hyperproliferative ECs is sufficient to cause an AVM.

Third, recent findings of somatic mutations leading to a second (bi-allelic) genetic hit in *ENG* or *ALK1* within vascular lesions from HHT1 and HHT2 patients, respectively is important (45). It is consistent with extensive animal model data showing requirement for bi-allelic loss of function for reproducible AVM formation. On the other hand, a recent study has shown that tissues such as skin, with high susceptibility to vascular lesions in HHT1, have reduced basal ENG levels compared with low risk tissues such as kidney and heart (115). In consequence, ENG proteins levels in *Eng*^+/−^ mice fall to almost undetectable levels in skin capillaries. Furthermore, early immunostaining data suggested the inherited “good” allele remained functional in HHT patient AVM tissue (116). These findings are consistent with the haploinsufficient model for HHT1, where a threshold level of ENG protein is required for it to function effectively and does not invoke a second genetic hit. However, this concept currently remains at odds with extensive animal model data showing requirement for bi-allelic loss of function for AVM formation. The disparity may be due to the insensitivity of immunostaining for detecting loss of function mutations, especially where there is mosaicism. In addition, prior transient loss of ENG protein due to cleavage of its extracellular domain resulting in an AVM is unlikely to be detectable at a later time point. Therefore, the contribution of EC-specific second hit mutations in HHT patient AVMs urgently needs to be clarified. Investigations using single cell (sc) DNA sequence analysis of HHT patient AVM tissues are ongoing, and will determine whether or not second genetic hits are present in a broader range of vascular lesions in HHT patients. These studies together with sc transcriptomics will also be extremely informative of gene expression changes within lesion ECs and their supporting cells and should reveal differences between HHT1 and HHT2 pathologies.

Fourth, although the requirement for an angiogenic trigger, either developmental or pathological, to drive AVM formation has been recognised for some time, evidence now suggests loss of *ENG* or *ALK1* function also generates abnormal EC responses to VEGF signalling. Targeting angiogenesis by reducing VEGF activity therefore has a dual beneficial effect and intravenous Bevacizumab is an effective and reasonably tolerated therapy in adult HHT patients. Further understanding of the molecular players downstream of VEGF signalling and how these responses are altered following loss of ENG/ALK1 signalling will allow refinement of future therapies in HHT.

Despite these advances, it still remains unclear what causes the tissue specific location of vascular lesions in HHT patients. Lung, skin, oronasal, and GI tract are all regularly exposed to environmental and/or inflammatory insults consistent with the hypothesis that postnatal development of AVMs in these tissues may be driven by inflammatory triggers. Local inflammation is also a normal part of liver homeostasis as this organ is exposed *via* the portal vein to microbial and other gut-derived antigenic factors (117). In HHT1 patients there is a higher incidence of pulmonary AVMs than in HHT2 patients. As a result there is an increased risk in HHT1 of microthrombi or bacteria bypassing the pulmonary capillaries to reach the cerebral circulation to act as pro-inflammatory triggers, which may explain why brain AVMs are more frequent in HHT1 patients than HHT2 patients. Whether HHT patients with brain AVMs have an increased propensity to have pulmonary AVMs (either symptomatic or clinically silent) requires investigation. On the other hand, cerebral AVMs in newborns are unlikely to result from exposure to unfiltered infectious agents and in these cases somatic mutation may be an underlying causal event.

It is also worth considering that telangiectases may result from reduced vasoconstriction capacity in naturally occurring arteriovenous anastomoses. In humans, these anastomoses are numerous in thermoregulated tissues such as mucosal tissues, glabrous skin (palms and soles of hands and feet) and nailbeds that are also frequent sites of telangiectases. Sympathetic nerves stimulate vasoconstriction of AV anastomoses to divert blood to skin surface capillaries to enable heat loss and maintain core temperature at 37°C. Reduced EC-VSMC crosstalk, as occurs when endothelial ENG levels are reduced (74, 76) may reduce the vasoconstriction efficacy of these AV anastomoses leading to increased arteriovenous shunting and the formation of visible telangiectases. Detailed longitudinal imaging studies of developing dermal telangiectases in HHT patients would help discriminate this possibility.

In conclusion, recent advances have uncovered much new evidence to explain AVM formation in HHT and inform effective therapies for patients (summarised in Table 1). For example, based on the preclinical models of HHT, increasing availability of transmembrane ALK1 protein would protect against AVM formation in both HHT1 and HHT2 patients. Furthermore, in principle, increased availability of ligand (BMP10) should provide protection against AVM formation in HHT1, whilst targeting EC mechanotransduction defects opens up a whole new therapeutic framework for investigation. In addition, improved EC-VSMC crosstalk would improve vessel stability and reduce bleeding. Of these potential approaches, only anti-VEGF targeting and thalidomide-related drugs are currently being used in the clinic to treat HHT, and their use predated our current conceptual understanding of their protective mechanisms in HHT. There is therefore a need to build on our recent advances in mechanistic insights to improve these therapies. Furthermore, although we have a better understanding of AVM formation, there is very little understanding about the options for promoting AVM regression. For example in the context of increased blood flow through abnormal shunts in HHT, it is not known which molecular targets could be used to normalise the vasculature or whether treatments simply to stabilise pre-existing vascular lesions provide the most realistic therapeutic options. However, ongoing work in preclinical models is rapidly moving towards translation and now that multiple models of HHT are available, further exciting advances in this area are anticipated sooner rather than later.

**TABLE 1:** Strategies to prevent AVMs in preclinical models of HHT.

Drug	Target	Model	Outcome	References
DC101	Anti-VEGFR2 antibody	Adult *Eng-iKOe* mice-pelvic AVMs and HOHF	Protection against AVMs and HOHF	(33)
DC101	Anti-VEGFR2 antibody	Adult *Alk1* ± mice with infected tracheas	Prevention of multiple telangiectases	(100)
G6.31	Anti-VEGFA antibody	Adult *Alk1-iKOe* mice with wound induced dermal AVMs	Prevention and possible reversal of established AVMs	(99)
SU5416	VEGFR2 inhibitor	Neonatal *Eng-iKOe* mice	Significant reduction in retinal AVM size	(46)
AV951	VEGFR2 inhibitor	Embryonic *eng* null Zebrafish	Prevents aortic and cardinal vein enlargement	(91)
Sorafenib and Pazopanib analogue (GW771806)	Tyrosine kinase inhibitors (TKI)	Adult *Alk1-iKOe* adult mice with wound-induced dermal AVMs	Each drug alone improved Hb and GI bleeding but did not prevent dermal AVMs	(118)
Wortmannin or Pictilisib	PI3K inhibitor	Neonatal *Eng-iKOe*, *Alk1-iKOe*, and *Smad4-*mice	Reduced retinal AVMs	(29, 46, 87)
Nintedanib and Sirolimus	TKI and mTOR inhibitor	Neonatal mice with antibody blockade of BMP9/10	Combination therapy reduced and potentially reversed retinal AVMs	(98)
PD0325901 + Rapamycin	Synergistic ERK + mTOR inhibitors	Embryonic *eng* null Zebrafish	Prevents aortic and cardinal vein enlargement	(91)
Thalidomide	Increased PDGFB expression	Adult *Eng* ± and *Alk1-iKO* mice	Increased muscle coverage of dermal and cerebral vessels; reduced cerebral haemorrhage	(76, 83)
LC10	ANGPT2 inhibitor	Neonatal *Smad4-iKOe* mice	Reduced retinal AVMs	(119)
Cilentgitide or ATN161	Integrin inhibitors	Neonatal *Alk1-iKOe* mice	Reduced retinal AVMs	(55)
Verteporfin	Yap/Taz inhibitor	Neonatal *Alk1-iKOe* mice	Reduced retinal AVMs	(55)
Tetrabromocinnamic acid or CX-4945	CK2 inhibitor	Neonatal *Smad4-iKOe* mice	Reduced retinal AVMs	(29)
Overexpression of ALK1	Genetic approach	Adult and neonatal ENG-iKO and ALK1-iKO mice	Rescues AVM formation in retina and dermal wound model	(14)

## Author contributions

HA wrote the first draft of the manuscript. BR expanded the initial draft. Both authors contributed to manuscript revision and approved the submitted version.
